# Identification of subtypes and construction of a predictive model for novel subtypes in severe community-acquired pneumonia based on clinical metagenomics: a multicenter, retrospective cohort study

**DOI:** 10.3389/fcimb.2025.1676502

**Published:** 2025-12-09

**Authors:** Shiyi Chen, Yongpo Jiang, Dongqing Lv, Ye Zheng, Ruihai Zhang, Hanzhi Dai, Ziyi Wang, Shuyang Li, Rongbin Qi, Hailing Xu, Yingying Yu, Cailin Xu, Xuanyu Lu, Yinghe Xu, Shengwei Jin, Xiaomai Wu

**Affiliations:** 1 Department of Respiratory Medicine, Taizhou Hospital of Zhejiang Province Affiliated to Wenzhou Medical University, Taizhou, China; 2 Department of Critical Care, Taizhou Hospital of Zhejiang Province Affiliated to Wenzhou Medical University, Taizhou, China; 3 Department of Anesthesia and Critical Care, The Second Affiliated Hospital and Yuying Children’s Hospital of Wenzhou Medical University, Wenzhou, China

**Keywords:** severe community-acquired pneumonia, pulmonary microbiome, metagenomic next-generation sequencing, machine learning, subtype classification

## Abstract

**Objective:**

It is well recognized that high heterogeneity represents a key driver of the elevated mortality in severe community-acquired pneumonia (sCAP). Precise subtype classification is therefore critical for both treatment strategy formulation and prognostic evaluation in this patient population. This study aimed to develop a predictive model for novel clinical subtypes of sCAP, leveraging microbiome profiles identified via metagenomic next-generation sequencing (mNGS).

**Methods:**

This retrospective multicenter cohort study enrolled adult patients with sCAP who underwent clinical mNGS testing of bronchoalveolar lavage fluid in intensive care units (ICUs) across 17 medical centers in China. Based on mNGS-identified microbiome characteristics, unsupervised machine learning (UML) was employed for clustering analysis of sCAP patients. LASSO regression and random forest (RF) algorithms were applied to screen and identify predictors of novel sCAP subtypes. A predictive model for the new clinical subtypes was constructed according to the screening results, with a nomogram generated. The discriminative ability, calibration, and clinical utility of the model were evaluated using ROC curves, calibration curves, and decision curve analysis, respectively.

**Results:**

A total of 1,051 sCAP patients were included in the final analysis. The 28-day all-cause mortality rate was 45% (473/1,051). UML clustering identified two distinct sCAP subtypes: the 28-day mortality rate was 42.19% (343/813) in subtype 1 and 54.62% (130/238) in subtype 2. Incorporating clinical and microbial features, a predictive model for the novel sCAP subtypes was developed using the following predictors: immunosuppression (OR = 37,411.46, P < 0.001), connective tissue disease (CTD) (OR = 12,144.60, P = 0.004), hematological malignancy (HM) (OR = 107,768.13, P < 0.001), chronic kidney disease (CKD) (OR = 49.71, P < 0.001), cytomegalovirus (CMV) (OR = 0.00, P < 0.001), Epstein-Barr virus (EBV) (OR = 131.97, P < 0.001), Pneumocystis (OR = 47,949.56, P < 0.001), and Klebsiella (OR = 0.02, P = 0.003). The model demonstrated excellent discriminative ability with an area under the ROC curve (AUC) of 0.992. Calibration curves showed good agreement between predicted and observed outcomes. Decision curve analysis confirmed high clinical utility for predicting novel sCAP subtypes.

**Conclusion:**

This study identified novel clinical subtypes of sCAP based on mNGS-derived microbiome characteristics. This approach exhibits superior performance in identifying high-risk sCAP patients, facilitating precise subtyping.

## 1 Introduction

Severe community-acquired pneumonia (sCAP) is among the leading causes of death from infectious diseases globally, with its incidence continuing to rise worldwide, especially in elderly individuals, those with comorbidities, and immunocompromised patients (Nair and Niederman, 2021; Huang et al., 2024). Hospital mortality in sCAP is high, ranging from 25% to over 50% (Niederman and Torres, 2022). Traditional classification systems, which rely solely on macro-level clinical indicators, fail to account for the marked heterogeneity and variability in mortality observed in sCAP patients. This indicates that current standards neglect critical sources of heterogeneity, highlighting the need for subtype analysis to identify potentially high-risk subgroups (Xu et al., 2023a; Peetermans et al., 2024; Jiang et al., 2025b). Thus, there is an urgent requirement for precise subtyping of sCAP patients, coupled with the implementation of individualized, stratified diagnosis and treatment strategies, to effectively improve patient outcomes and alleviate the healthcare burden.

In recent years, machine learning (ML) has advanced significantly in the field of pneumonia prognosis prediction, with particular value demonstrated in subtype identification and risk stratification. The XGBoost model by Xu et al. accurately predicts adverse outcomes in sCAP patients through integration of 12 clinical features (Xu et al., 2022). The Qin team further showed that a random forest model constructed using inflammatory markers and immune indicators can predict 30-day mortality and severity classification in CAP patients (Qin et al., 2024). However, tools currently used in clinical practice for predicting sCAP prognosis are primarily derived from routine clinical indicators, such as underlying diseases, inflammatory markers, and imaging features (Fourgeaud et al., 2024). These tools fail to capture the critical role of the pulmonary microbiome in sCAP prognosis, resulting in substantial population bias in their clinical application and limiting their ability to accurately predict outcomes across all patient groups.

Metagenomic next-generation sequencing (mNGS), as a first-line diagnostic tool, significantly enhances pathogen identification rates in sCAP patients while enabling the acquisition of respiratory tract microbiome data (Xu et al., 2023b; Fourgeaud et al., 2024; Wu et al., 2025). In microbiome research, mNGS overcomes the limitation of targeted sequencing methods, which are restricted to studying only bacteria or fungi, allowing for efficient analysis of all microorganisms in the lungs (Miao et al., 2018). More importantly, mNGS provides a comprehensive analysis of the true ecological structure of the pulmonary microbiome, encompassing low-abundance flora and difficult-to-culture pathogens, thereby better capturing the dynamic balance or disordered state of respiratory microorganisms. These microbial characteristics are closely linked to the host’s immune status, with colonization by opportunistic pathogens often indicating impaired immune function (Vidaur et al., 2025). Furthermore, viral co-activation has been confirmed to be associated with poor prognosis (Liu et al., 2025). Thus, establishing subtyping and prognostic tools for pulmonary microbiome analysis based on mNGS can better reflect the mechanisms underlying the occurrence and progression of sCAP, and offer more accurate information for prognostic assessment of sCAP.

Given high heterogeneity is a key factor contributing to elevated mortality in severe Community-Acquired Pneumonia (sCAP), the accurate subtype classification of patients with sCAP is essential for formulating treatment strategies and performing prognostic assessment. However, the specific microbial changes associated with sCAP and their contributions to disease severity remain unclear. To address this gap, this study aims to identify different clinical subtypes of sCAP using metagenomic next-generation sequencing (mNGS) and develop a predictive model based on microbiome characteristics. This approach is expected to provide a more detailed understanding of sCAP heterogeneity and improve the accuracy of prognostic evaluation. We hypothesize that integrating relevant clinical data and microbiome data will reveal novel sCAP subtypes with unique microbiome characteristics, and these subtypes may be associated with disease severity and mortality.

## 2 Material and methods

### 2.1 Study design and patient enrollment

This multicenter, retrospective cohort study was conducted in adult intensive care units (ICUs) across 17 medical centers in China, including clinical data and mNGS results of all sCAP patients admitted to the ICU from January 1, 2019, to June 30, 2023. Inclusion criteria were: 1. Age ≥ 18 years, 2. Diagnosis of severe community-acquired pneumonia (sCAP), 3. Undergoing commercial metagenomic next-generation sequencing (mNGS) of bronchoalveolar lavage fluid. The exclusion criterion was: 1. Lost to follow-up or abandonment of treatment within 28 days after ICU admission, 2. Patients with concurrent COVID-19 infection. This study was approved by the ethics committees of all participating hospitals (Approval Number: K20230510). Informed consent was waived due to the use of retrospective data.

### 2.2 Definition and data collection

The diagnosis of sCAP is based on the criteria for assessing CAP severity established by the Infectious Diseases Society of America (IDSA) and the American Thoracic Society (ATS). Diagnosis requires meeting one of two major criteria or three or more minor criteria. Major criteria are: requiring mechanical ventilation via endotracheal intubation, (2) needing vasoactive agents following aggressive fluid resuscitation for septic shock. Minor criteria are: (1) respiratory rate ≥ 30 breaths/min, (2) PaO_2_/FiO_2_ ≤ 250 mm Hg, (3) multilobar infiltrates, (4) altered mental status and/or disorientation, (5) blood urea nitrogen ≥ 20 mg/dL. Immunosuppression is defined as previously described: (1) neutropenia (< 0.5 × 109/L for ≥ 10 days post-admission), (2) recent use of immunosuppressants (e.g., tacrolimus, cyclosporine, rituximab), or (3) history of AIDS, or organ transplantation.

All clinical metagenomic tests were performed in accordance with previously reported protocols (Huang et al., 2023), with results available within 36 hours of sample submission. During clinical metagenomic testing, all patients or their families were informed of relevant details and signed informed consent forms for the tests in accordance with Chinese law. For patients who underwent multiple clinical metagenomic tests, only results from the first test were included in the analysis.

Data were collected in a standardized manner via review of electronic medical record systems. Collected data included gender, age, comorbidities, immunosuppressive status, laboratory test results, clinical metagenomic results, and clinical scores (Sequential Organ Failure Assessment [SOFA]) for included patients. Data collection across all centers adhered to uniform standards. The clinical metagenomic laboratory was accredited by the College of American Pathologists or the external quality assessment program of the National Health Commission of China.

### 2.3 Statistical analysis

Statistical analyses in this study were performed using SPSS 27.0 software. Measurement data with a normal distribution were expressed as mean ± standard deviation; those without a normal distribution were expressed as median (interquartile range, Q1-Q3). Group comparisons for normally distributed data, non-normally distributed data, and categorical data used the Student’s t-test, Mann-Whitney test (for skewed data), and Pearson’s chi-square test (for categorical variables), respectively. The level of statistical significance was set at P < 0.05.

We used R software (v4.1.3) for unsupervised machine learning (UML) analysis. Data from 1051 sCAP patients were normalized using the “factoextra” package in R. Patients were clustered via the K-means algorithm within UML. The “Fpc” package was used to calculate silhouette coefficients for determining the optimal number of clusters, with cluster plots employed to visualize K-means clustering results. Survival analysis was performed using Kaplan-Meier curves.

This study used LASSO regression to screen predictive variables. LASSO regression analysis was performed via importing collected data into R software (using the “glmnet” package) to identify predictive factors for novel sCAP subtypes. Concurrently, the random forest (RF) algorithm was used to identify predictive factors for novel sCAP subtypes, with a 5-time 10-fold cross-validation iterative process to reduce overfitting risk. A predictive model for novel sCAP subtypes was then constructed using the forward selection method in logistic regression analysis, with collinearity testing conducted concurrently. The model was visually presented via a nomogram. Additionally, the “pROC” and “ggplot2” packages in R were used to plot receiver operating characteristic (ROC) curves and evaluate model discriminative ability. The “rmst” package was used to generate calibration curves. The “dcurves” and “rmda” packages were used to plot decision curves for assessing the clinical benefits of the model.

## 3 Results

### 3.1 Patient characteristics

**Figure 1:**
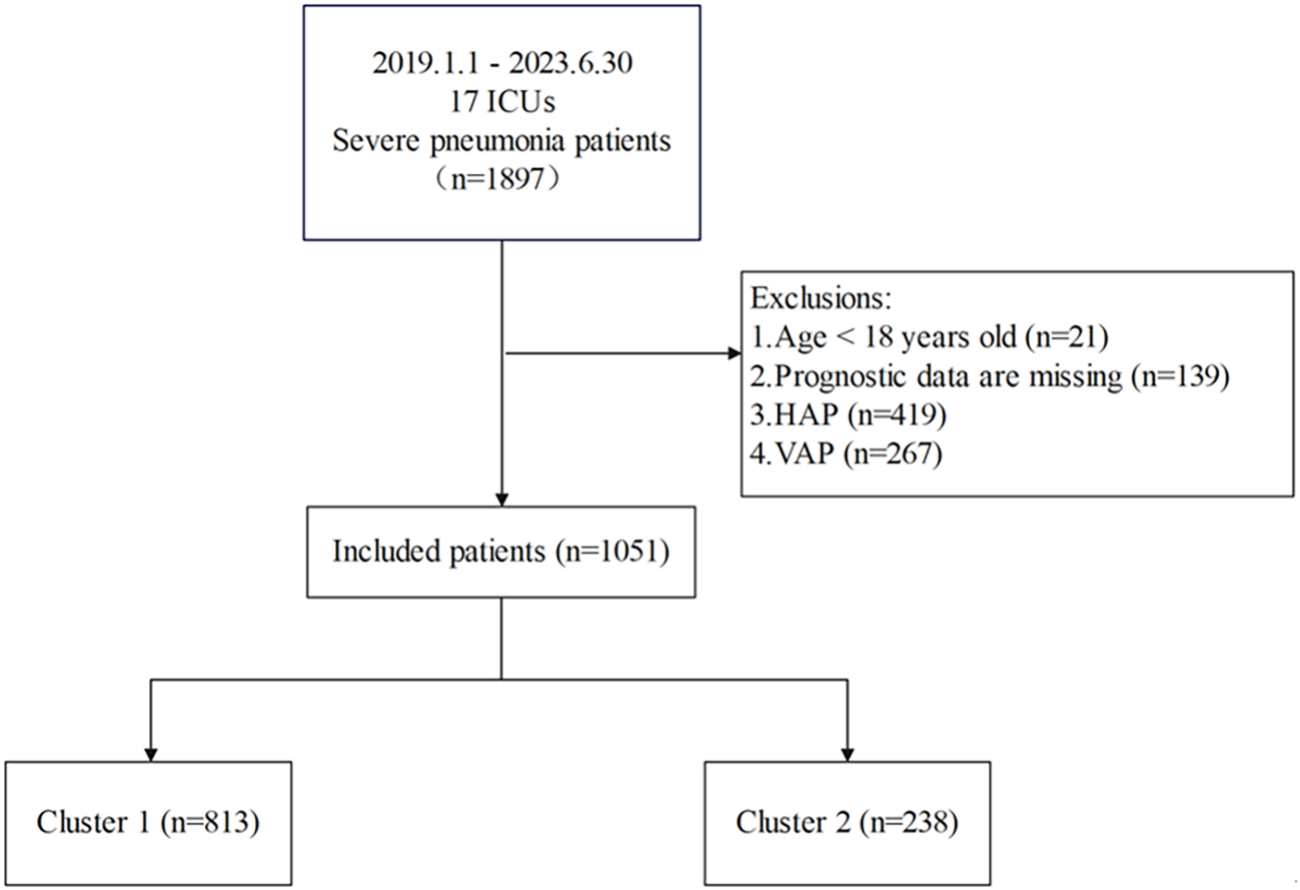
Inclusion and exclusion criteria flowchart.

**Figure 2:**
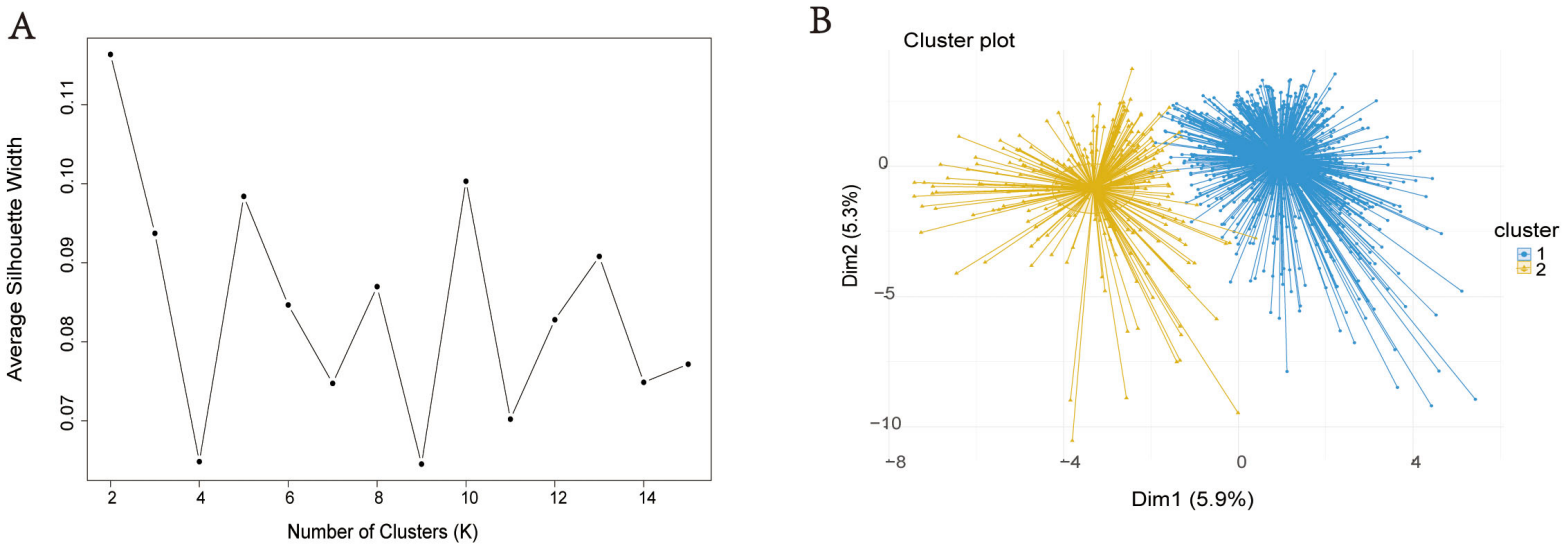
Visualization of unsupervised machine learning. Silhouette Coefficient (SC) of K-means clustering algorithm which was determined the optimal clustering result. Peak of the broken line is the optimal value for Silhouette Coefficient (Y Axis), the optimal clustering results were equal to 2 (X Axis) **(A)**. Scatter plots of 1051 sCAP patients. Each dot in the figure represents a patient. The blue scatter represents cluster 1 and the yellow scatter represents cluster 2 **(B)**.

This study used LASSO regression to screen predictive variables: A total of 1,897 patients with severe pneumonia admitted to adult intensive care units were screened, with 1,051 finally included after excluding 21 patients aged < 18 years, 139 with missing prognostic data, 419 with hospital-acquired pneumonia, and 267 with ventilator-associated pneumonia (**Figure 1**). Patients were divided into 2 clusters via unsupervised machine learning: Cluster 1 (n = 813) and Cluster 2 (n = 238) (**Figure 2**). Among the 1,051 included patients, 69.27% (728/1,051) were male, with a mean age of 65.06 ± 16.06, and the 28-day mortality rate was 45% (473/1,051). **Table 1** presents comparisons of baseline data between the two clusters, including demographics, comorbidities, laboratory indicators, and metagenomic data. The median age of Cluster 1 was 68.00 (57.00, 76.00), with 71.34% male. The median age of Cluster 2 was 64.50 (55.00, 73.00), with 62.18% male. The 28-day mortality rate was 42.19% (343/813) in Cluster 1 and 54.62% (130/238) in Cluster 2, with a statistically significant difference between the two clusters (P < 0.001). Compared with Cluster 1, Cluster 2 had a higher proportion of immunosuppression, as well as higher incidences of liver disease, chronic kidney disease (CKD), hematologic malignancy(HM), connective tissue disease (CTD), and a higher rate of transplantation history (Immunosuppression: Cluster 1/Cluster 2 = 9.72%/83.19%, p < 0.001; Liver Disease: Cluster 1/Cluster 2 = 5.04%/10.5%, p = 0.002; CKD: Cluster 1/Cluster 2 = 8.36%/34.03%, p = 0.002; HM: Cluster 1/Cluster 2 = 0.49%/16.39%, p < 0.001; CTD: Cluster 1/Cluster 2 = 0.98%/19.33%, p < 0.001; Transplantation: Cluster 1/Cluster 2 = 0.00%/23.95%, p < 0.001).

**Table 1:** Characteristics of patients of the two clusters.

Characteristics	Cluster 1 (n = 813)	Cluster 2 (n = 238)	P
Age, years, median (IQR)	68.00 (57.00, 76.00)	64.50 (55.00, 73.00)	<.001
Male gender, n(%)	580 (71.34)	148 (62.18)	0.007
Laboratory tests, median (IQR)			
White blood cell, 10^9^/L	11.40 (7.05, 16.30)	10.61 (6.20, 15.04)	0.045
Lymphocyte, 10^9^/L	0.58 (0.32, 0.94)	0.43 (0.24, 0.81)	<.001
Neutrophil, 10^9^/L	10.03 (5.79, 14.40)	9.07 (5.00, 13.63)	0.057
C reactive protein, mg/L	98.62 (40.60, 170.46)	89.01 (36.11, 160.02)	0.070
procalcitonin, ng/L	1.00 (0.22, 6.78)	0.78 (0.25, 4.28)	0.245
Intravenous Corticosteroids, n(%)	380 (46.74)	39 (16.39)	<.001
Comorbidities, n(%)			
Diabetes	226 (27.80)	54 (22.69)	0.117
Myocardial infarction	48 (5.90)	12 (5.04)	0.614
Chronic pulmonary disease	174 (21.40)	56 (23.53)	0.485
Liver disease	41 (5.04)	25 (10.50)	0.002
Chronic kidney disease	68 (8.36)	81 (34.03)	<.001
Solid tumor	103 (12.67)	39 (16.39)	0.140
Hematologic malignancy	4 (0.49)	39 (16.39)	<.001
Connective tissue disease	8 (0.98)	46 (19.33)	<.001
Transplantation	0 (0.00)	57 (23.95)	<.001
Cerebrovascular disease	147 (18.08)	28 (11.76)	0.021
Immunosuppression, n(%)	79 (9.72)	198 (83.19)	<.001
Clinical metagenomics results, n (%)			
Acinetobacter spp.	237 (29.15)	43 (18.07)	<.001
Klebsiella spp.	256 (31.49)	28 (11.76)	<.001
Pseudomonas spp.	108 (13.28)	29 (12.18)	0.658
Stenotrophomonas spp.	103 (12.67)	31 (13.03)	0.885
Enterococcus spp.	129 (15.87)	41 (17.23)	0.616
Burkholderia spp.	62 (7.63)	10 (4.20)	0.066
Staphylococcus spp.	87 (10.70)	15 (6.30)	0.044
Corynebacterium spp.	760 (93.48)	228 (95.80)	0.185
Escherichia spp.	37 (4.55)	6 (2.52)	0.164
Streptococcus spp.	93 (11.44)	12 (5.04)	0.004
Haemophilus spp.	45 (5.54)	5 (2.10)	0.029
Elizabethkingia spp.	25 (3.08)	6 (2.52)	0.657
Achromobacter spp.	19 (2.34)	9 (3.78)	0.224
Enterobacter spp.	18 (2.21)	5 (2.10)	0.916
Candida spp.	254 (31.24)	71 (29.83)	0.679
Pneumocystis spp.	16 (1.97)	104 (43.70)	<.001
Aspergillus spp.	119 (14.64)	65 (27.31)	<.001
Torque Teno Virus	58 (7.13)	43 (18.07)	<.001
Nakaseomyces spp.	52 (6.40)	8 (3.36)	0.076
Serratia spp.	21 (2.58)	2 (0.84)	0.106
HSV-1	200 (24.60)	65 (27.31)	0.397
EBV	98 (12.05)	92 (38.66)	<.001
CMV	78 (9.59)	113 (47.48)	<.001
HHV-7	32 (3.94)	18 (7.56)	0.021
HHV-6b	8 (0.98)	13 (5.46)	<.001
Duration of mechanical ventilation within 28 days, days, median (IQR)	8.00 (3.00, 14.00)	7.00 (2.00, 12.00)	0.073
SOFA score at transfer to ICU, median (IQR)	7.00 (4.00, 9.00)	7.00 (5.00, 10.00)	0.168
28-day mortality, n (%)	343 (42.19)	130 (54.62)	<.001

Data are presented as median (interquartile range), n (%).

HSV, Herpes simplex virus; EBV, Epstein-Barr virus; CMV, Cytomegalovirus; HHV, Human herpes virus; IQR, interquartile range; ICU, Intensive Care Unit; SOFA, Sequential Organ Failure Assessment.

Additionally, the proportions of infections with Pneumocystis, Aspergillus, Torque Teno Virus, Epstein-Barr virus (EBV), Cytomegalovirus (CMV), human herpesvirus 7 (HHV-7), and human herpesvirus 6b (HHV-6b) were higher (Pneumocystis: Cluster 1/Cluster 2 = 1.97%/43.70%, p < 0.001; Aspergillus: Cluster 1/Cluster 2 = 14.64%/27.31%, p < 0.001; Torque Teno Virus: Cluster 1/Cluster 2 = 18.07%/7.03%, p < 0.001; EBV: Cluster 1/Cluster 2 = 12.05%/38.66%, p < 0.001; CMV: Cluster 1/Cluster 2 = 9.59%/47.48%, p < 0.001; HHV-7: Cluster 1/Cluster 2 = 3.94%/7.56%, p < 0.001; HHV-6b: Cluster 1/Cluster 2 = 5.46%/0.98%, p < 0.001) (**Table 2**). In contrast, Cluster 1 had higher levels of white blood cells (WBC) and lymphocytes, a higher incidence of cerebrovascular disease (CBD) (WBC: Cluster 1/Cluster 2 = 11.40 (7.05, 16.30)/10.61 (6.20, 15.04), p = 0.045; Lymphocyte: Cluster 1/Cluster 2 = 0.58 (0.32, 0.94)/0.43 (0.24, 0.81), p < 0.001; CBD: Cluster 1/Cluster 2 = 18.08%/11.76%, p < 0.001), as well as higher proportions of infections with Acinetobacter, Klebsiella, Staphylococcus, Streptococcus, and Haemophilus (Acinetobacter: Cluster 1/Cluster 2 = 29.15%/18.07%, p < 0.001; Klebsiella: Cluster 1/Cluster 2 = 31.49%/11.76%, p < 0.001; Staphylococcus: Cluster 1/Cluster 2 = 10.70%/6.30%, p < 0.001; Streptococcus: Cluster 1/Cluster 2 = 11.44%/5.04%, p = 0.004; Haemophilus: Cluster 1/Cluster 2 = 5.54%/2.10%, p = 0.029).

**Table 2:** The results of univariate and multivariate logistic regression.

Variables	Univariate logistic regression		Multivariate logistic regression	
	*P*-value	OR (95%CI)	*P*-value	OR (95%CI)
Immunosuppression	<0.001	45.99 (30.48 ~ 69.40)	<0.001	37411.46 (880.14 ~ 1590228.22)
Transplantation	0.971	191099074.13 (0.00 ~ Inf)	0.984	2233694628614.21 (0.00 ~ Inf)
Intravenous Corticosteroids	<0.001	0.22 (0.15 ~ 0.32)	0.052	0.17 (0.03 ~ 1.01)
HM	<0.001	39.64 (14.00 ~ 112.19)	<0.001	107768.13 (1124.57 ~ 10327465.93)
CTD	<0.001	24.11 (11.19 ~ 51.92)	0.004	12144.60 (20.55 ~ 7178326.55)
CKD	<0.001	5.65 (3.92 ~ 8.15)	<0.001	49.71 (9.25 ~ 267.23)
CMV	<0.001	0.12 (0.08 ~ 0.17)	<0.001	0.00 (0.00 ~ 0.02)
EBV	<0.001	4.60 (3.29 ~ 6.43)	<0.001	131.97 (19.78 ~ 880.33)
Klebsiella	<0.001	0.29 (0.19 ~ 0.44)	0.003	0.02 (0.00 ~ 0.25)
Pneumocystis	<0.001	38.66 (22.14 ~ 67.50)	<0.001	47949.56 (1078.38 ~ 2132047.86)

HM, Hematologic malignancy; CTD, Connective tissue disease; CKD, Chronic kidney disease; CMV, Cytomegalovirus; EBV, Epstein-Barr virus.

### 3.2 Survival curve analysis

**Figure 3:**
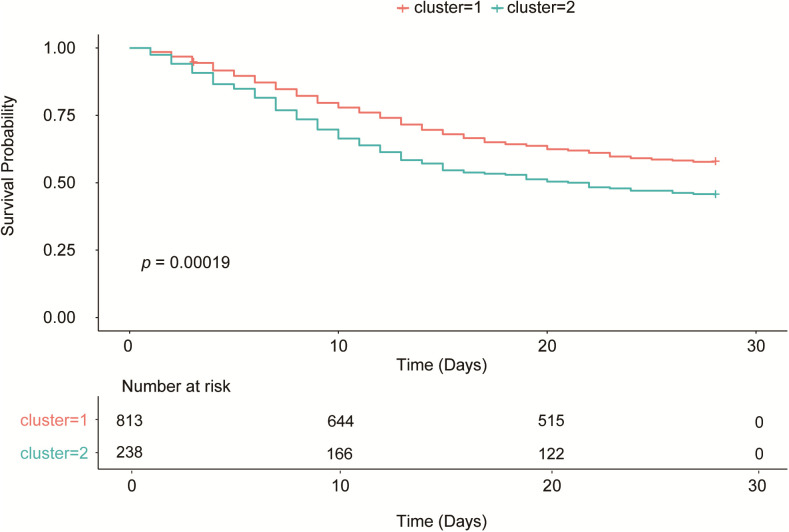
Kaplan-Meier curves of the clusters.

In the survival analysis, the Kaplan-Meier curve (**Figure 3**) showed that the survival rate of patients in Cluster 2 was significantly lower than that in Cluster 1 (p = 0.00019). The 28-day mortality rate was 42.19% (343/813) in Cluster 1 and 54.62% (130/238) in Cluster 2, with a statistically significant difference between the two clusters (P < 0.001) (**Table 1**). Thus, Cluster 1 was defined as SCAPML1 and Cluster 2 as SCAPML2.

### 3.3 Factors associated WITH SCAPML2 and prediction model

**Figure 4:**
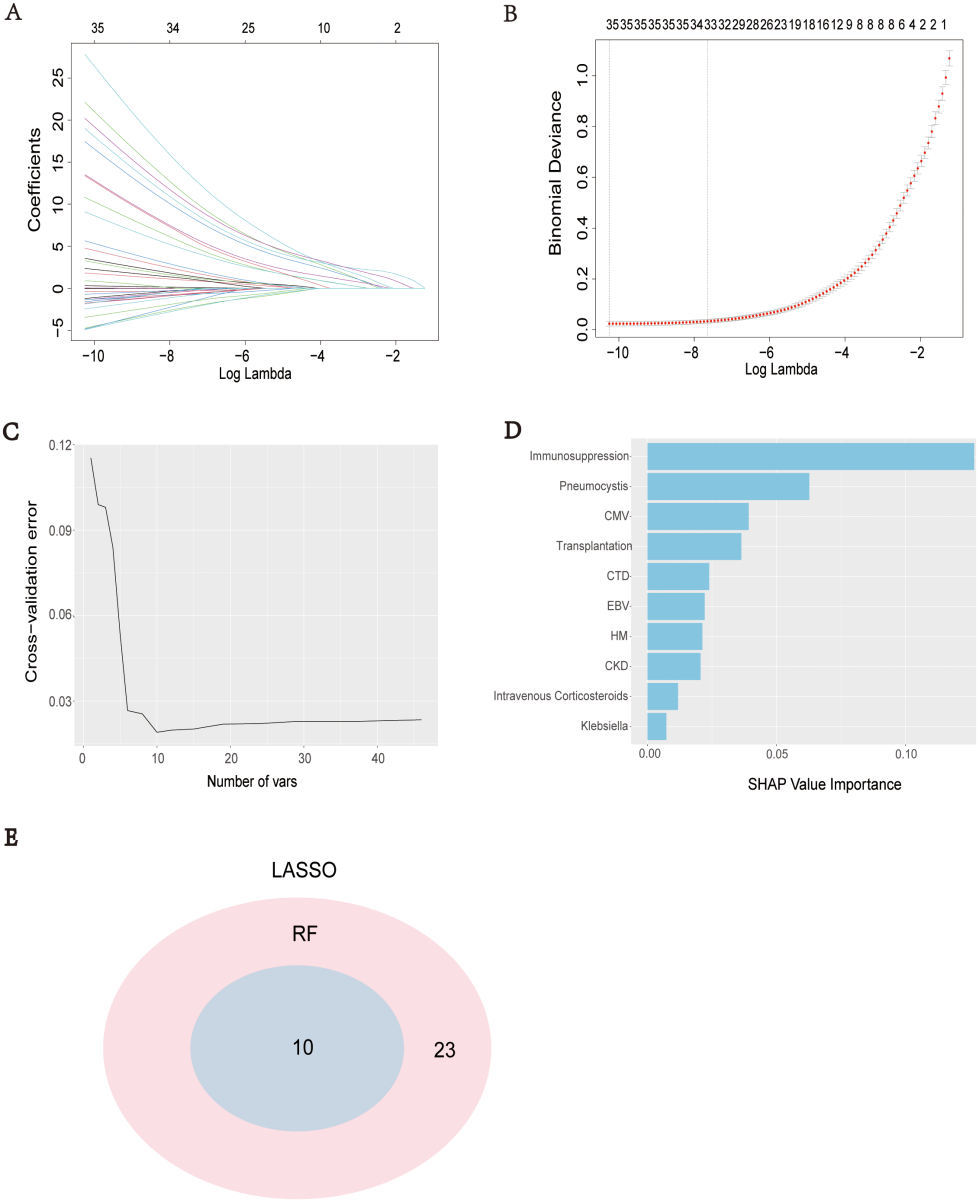
Selection of predictive factors. LASSO coefficient profiles of the predictive factors **(A)**, The results of LASSO regression **(B)**, The optimal regression results can be obtained by retaining the 10 predictive factors after five iterations of 10-fold cross validation **(C)**, 10 predictive factors were screened by IncNodePurity of Ramdon Forest algorithms **(D)**, Intersection of predictive factors screened using LASSO regression and Random Forest algorithm **(E)**.

LASSO regression analysis was performed based on clinical data and microbiome results to screen for predictive factors of SCAPML2, with 33 predictive factors finally identified, including Intime SOFA, Solid tumor, HM, CTD, Transplantation, CBD, Neutrophil, and Liver disease (**Figures 4A, B**). Concurrently, based on 5-time 10-fold cross-validation results (**Figure 4C**), the RF algorithm was used to screen for predictive factors of SCAPML2 (**Figure 4D**), identifying 10 key predictive factors: Immunosuppression, HM, CTD, CKD, CMV, Transplantation, EBV, Intravenous Corticosteroids, Klebsiella, and Pneumocystis. **Figure 4E** shows the intersection of predictive factors screened via LASSO regression and the RF algorithm, namely Immunosuppression, HM, CTD, CKD, CMV, Transplantation, EBV, Intravenous Corticosteroids, Klebsiella, and Pneumocystis.

Univariate and multivariate logistic regression analyses were performed on the 10 screened predictive factors, identifying 8 independent predictive factors: Immunosuppression (OR = 37411.46, P < 0.001), CTD (OR = 12144.60, P = 0.004), HM (OR = 107768.13, P < 0.001), CKD (OR = 49.71, P < 0.001), CMV (OR = 0.00, P < 0.001), EBV (OR = 131.97, P < 0.001), Pneumocystis (OR = 47949.56, P < 0.001), and Klebsiella (OR = 0.02, P = 0.003) (**Table 2**). Collinearity analysis was applied to assess collinearity among variables, with results showing all included variables had a VIF < 10 and Tolerance > 0.1, indicating no significant collinearity in the model (**Supplementary Table S1**).

### 3.4 Model evaluation and validation

**Figure 5:**
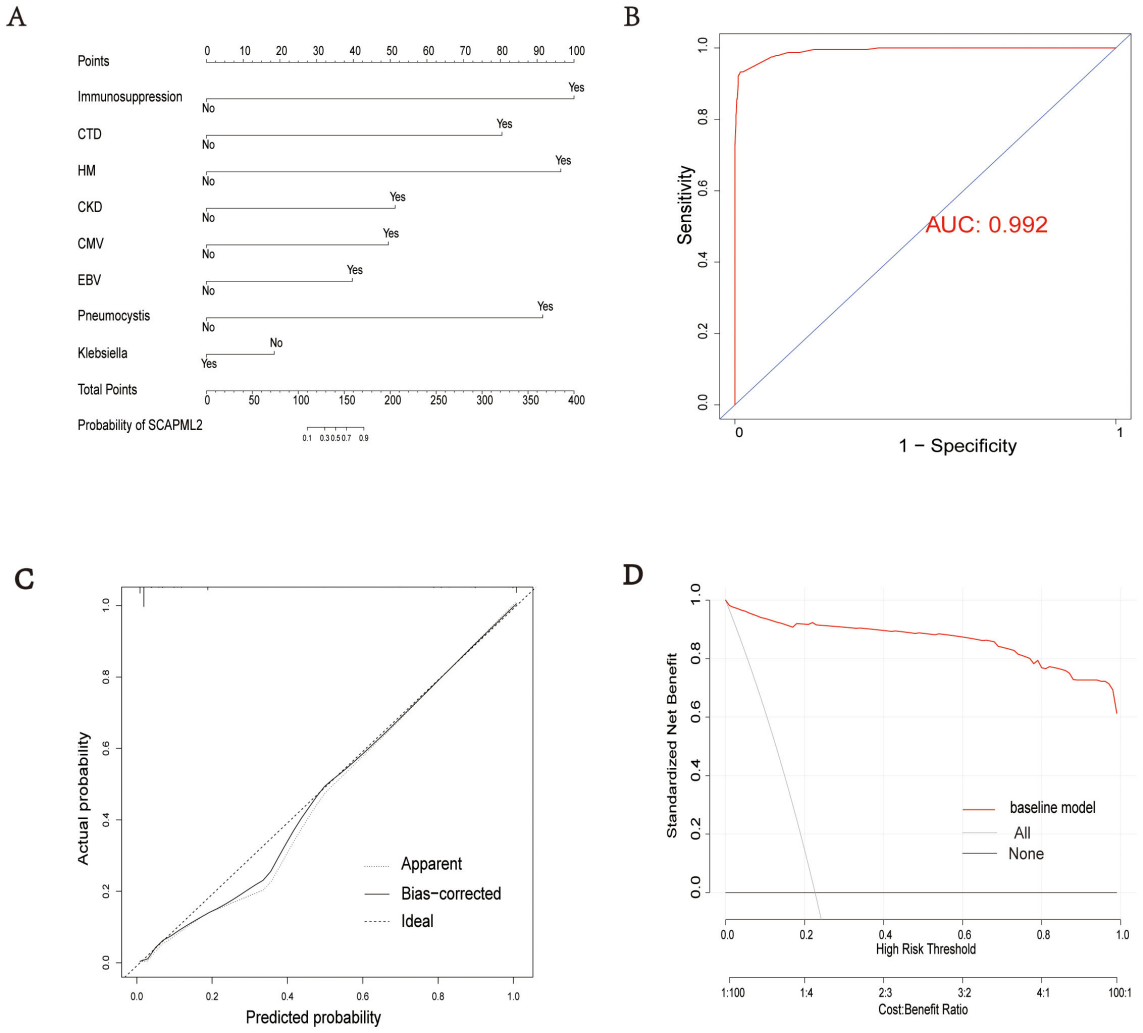
Model evaluation. Establishment of a nomogram for SCAPML2 **(A)**. AUC of the nomogram based on 8 predictive factors **(B)**. Calibration curves for predicting SCAPML2 probability **(C)**. Decision curves for model **(D)**.

A nomogram for predicting SCAPML2 was constructed based on the key factors screened by logistic regression analysis (**Figure 5A**). The diagnostic efficacy of the predictive model was evaluated using the receiver operating characteristic (ROC) curve, with an area under the curve (AUC) of 0.992 (**Figure 5B**), indicating good predictive performance. The calibration of the model was assessed via the calibration curve (Bootstrap method, n = 1000) (**Figure 5C**), and the Hosmer-Lemeshow test showed that the final model had a good fit (χ² = 0.628, P = 1.000). Decision curve analysis (DCA) with 1000 Bootstrap resamplings showed that the model had a high net benefit for predicting novel sCAP subtypes within the threshold probability range of 0.05–0.95 (**Figure 5D**).

## 4 Discussion

To our knowledge, this study is the first to apply unsupervised machine learning (UML) to the clinical subtyping of patients with severe community-acquired pneumonia (sCAP) by integrating microbiome characteristics derived from metagenomic next-generation sequencing (mNGS). Using this methodology, we successfully delineated a novel subtype (SCAPML2), and identified its independent predictive factors. The overall mortality rate of included sCAP patients was 45%, which is consistent with the mortality rates reported in previous studies (Garnacho-Montero et al., 2018; Martin-Loeches et al., 2025; Reyes et al., 2025). SCAPML2 is characterized by immunosuppression, specific comorbidities, and specific pathogenic infections, with a mortality rate as high as 54.62%, which is significantly higher than the 43% mortality rate of sCAP patients reported in prior research (Reyes et al., 2025). This subtype exhibits superior performance in identifying high-risk sCAP patients, highlighting the elevated risks faced by patients with this subtype.

The predictive model for SCAPML2 established in this study has a clear clinical early warning value. A German cohort study showed that immunosuppressed patients have a higher incidence of CAP and poorer prognosis (Reichel et al., 2024), which is consistent with our findings. Furthermore, research has shown that patients with hematological malignancies(HM) constitute a high-risk group for sCAP, and these patients are often complicated by infections with cytomegalovirus(CMV), Epstein-Barr virus(EBV), and Pneumocystis (Certan et al., 2022; Park et al., 2024; Jiang et al., 2025a). Additionally, literature has proposed that connective tissue disease-associated interstitial lung disease (CTD-ILD) is a common complication of CTD, and such patients typically receive long-term treatment with glucocorticoids or immunosuppressants, which increases the risk of pulmonary infections (Guiot et al., 2024). A study specifically constructed a 90-day mortality prediction model for CTD patients with pneumonia, showing that these patients have a higher risk of mortality (Li et al., 2023). Another study observed that in patients with chronic kidney disease (CKD) who have severe pneumonia, the incidence of acute kidney injury is significantly increased (OR = 2.45) and is associated with a higher risk of death (Xie et al., 2024). Meanwhile, this study identified CMV, EBV, and Pneumocystis as independent risk factors for predicting SCAPML2. Literature clearly indicates that among severe pneumonia patients, CMV, HSV-1, and EBV are the most common reactivated viruses, and their reactivation significantly increases the risk of death, specifically a 2.052-fold increase in 28-day mortality, suggesting a significant association between reactivation of these viruses and elevated death risk (Huang et al., 2023). Moreover, a multicenter prospective study indicated that microbial markers, including Pneumocystis jirovecii and viral markers such as EBV and CMV, correlate with poor prognosis (Song et al., 2025).

Notably, although Klebsiella is a typical pathogen in immunosuppressed patients (Calderón-Parra et al., 2025; Reyes et al., 2025), and tends to cause pneumonia, urinary tract infections, and bacteremia as a risk factor for poor prognosis (Alishvandi et al., 2025), the present study shows that Klebsiella negativity is significantly associated with a high risk of SCAPML2. This reflects the unique characteristics of this subtype, where immunosuppression, as an independent predictive factor, is present in a high proportion (83.19%). Studies have noted that sCAP patients with immunosuppression have multiple comorbidities, high drug resistance rates, and limited available antibiotics, thus supporting early use of potent antibiotics to cover potential high-risk pathogens (Ramirez et al., 2020). We hypothesize that such patients received potent antibiotics before disease progression to sCAP, which would significantly suppress infections with typical bacteria like Klebsiella. Meanwhile, immunosuppressive status represents a major risk factor for fungal and viral infections, as immune system impairment enables viruses and fungi to become dominant pathogens through immune evasion, increasing the risk of such infections (Reichel et al., 2024; Sayson et al., 2024). Taken together, heightened vigilance for the SCAPML2 subtype is needed in sCAP patients with evidence of immunosuppression, fungal or viral infection, and negativity for typical bacteria such as Klebsiella. Additionally, based on the clinical early warning value of the SCAPML2 predictive model, treatment strategies should comprehensively consider patients’ immune status, infecting pathogen types, and potential drug resistance risks to implement individualized therapy.

This study has several notable strengths. First, it is the first to integrate mNGS-based microbiome characteristics. With its unbiased full microbial coverage and high sensitivity, mNGS can efficiently analyze the overall ecological characteristics of the microbiome, thereby overcoming the inherent limitations of traditional detection methods in pathogen coverage, detection timeliness, and ecological perspective analysis (Simner et al., 2018). It also takes the lead in proposing a predictive model for a new subtype of sCAP, which shows good discriminative ability and calibration performance, providing a more comprehensive microbiome perspective for the precise diagnosis and treatment of sCAP. Second, we combined unsupervised machine learning (UML) for typing sCAP patients. This method can accurately classify patients according to the inherent heterogeneity of their clinical and microbiome characteristics, avoiding reliance on predefined labels in traditional supervised learning and thus more truly revealing the complexity of the patient population (Sajda, 2006). Additionally, the independent predictive factors identified and the predictive model constructed in this study provide clinicians with a tool for early identification of high-risk sCAP patient subtypes, facilitating timely adjustment of treatment plans, improvement of treatment effects, and reduction of mortality.

This study has several limitations. First, this study adopted a retrospective design, which inevitably introduces potential biases. On the one hand, data collection relied on the electronic medical record (EMR) system, in which information recording may be incomplete or may lack consistent standardization—both of which can introduce information bias. On the other hand, the retrospective design precludes random assignment of participants, which may give rise to selection bias driven by factors such as the timing of hospital admission and clinical testing conditions. Collectively, these limitations restrict the study’s ability to infer causal relationships. Second, mNGS primarily reflects the relative composition of microbial communities and cannot accurately quantify the absolute abundance of individual species. Additionally, it fails to distinguish between pathogenic infection and colonization. This limitation may obscure the microbiological basis for subtyping and thus necessitates comprehensive assessment in conjunction with clinical symptoms and imaging findings. Third, Third, the present study only conducted internal validation using data from a single-region cohort, and the constructed prediction model has not been validated in an independent external cohort. This shortcoming may reduce the applicability of the prediction model constructed in this study to other populations and limit the generalizability of the results. Future multicenter prospective studies should enroll patients with severe community-acquired pneumonia (sCAP) from diverse regions and populations, with the aims of validating the model externally, further optimizing its parameters, and enhancing its value for clinical translation.

## 5 Conclusion

This study identified a novel high-risk subtype, SCAPML2, in sCAP patients using unsupervised machine learning. Its independent predictive factors include immunosuppression, hematologic malignancies, and specific pathogen infections. The predictive model exhibits excellent discriminative efficacy, providing an important basis for the precise diagnosis and treatment of sCAP.

## Data Availability

The raw data supporting the conclusions of this article will be made available by the authors, without undue reservation.

## References

[B1] Alishvandi A. Barancheshemeh M. Firuzpour F. Aram C. Kamali M. J. Keikha M. (2025). Decoding virulence and resistance in Klebsiella pneumoniae: Pharmacological insights, immunological dynamics, and in silico therapeutic strategies. Microb. Pathog. 205, 107691. doi: 10.1016/j.micpath.2025.107691, PMID: 40355055

[B2] Calderón-Parra J. Carretero-Henriquez M. T. Escudero G. Suances-Martin E. Murga de la Fuente M. González-Merino P. . (2025). Current epidemiology, risk factors and influence on prognosis of multidrug resistance in Klebsiella spp. bloodstream infection. Insights from a prospective cohort. Eur. J. Intern. Med 139, 106369. doi: 10.1016/j.ejim.2025.05.034, PMID: 40506298

[B3] Certan M. Garcia Garrido H. M. Wong G. Heijmans J. Grobusch M. P. Goorhuis A. (2022). Incidence and predictors of community-acquired pneumonia in patients with hematological cancers between 2016 and 2019. Clin. Infect. Dis. 75, 1046–1053. doi: 10.1093/cid/ciac005, PMID: 35195716 PMC9522390

[B4] Fourgeaud J. Regnault B. Ok V. Da Rocha N. Sitterlé É. Mekouar M. . (2024). Performance of clinical metagenomics in France: a prospective observational study. Lancet Microbe 5, e52–e61. doi: 10.1016/S2666-5247(23)00244-6, PMID: 38048804

[B5] Garnacho-Montero J. Barrero-García I. Gómez-Prieto M. D. G. Martín-Loeches I. (2018). Severe community-acquired pneumonia: current management and future therapeutic alternatives. Expert Rev. Anti Infect. Ther. 16, 667–677. doi: 10.1080/14787210.2018.1512403, PMID: 30118377

[B6] Guiot J. Miedema J. Cordeiro A. De Vries-Bouwstra J. K. Dimitroulas T. Søndergaard K. . (2024). Practical guidance for the early recognition and follow-up of patients with connective tissue disease-related interstitial lung disease. Autoimmun. Rev. 23, 103582. doi: 10.1016/j.autrev.2024.103582, PMID: 39074630

[B7] Huang L. Zhang X. Pang L. Sheng P. Wang Y. Yang F. . (2023). Viral reactivation in the lungs of patients with severe pneumonia is associated with increased mortality, a multicenter, retrospective study. J. Med. Virol. 95, e28337. doi: 10.1002/jmv.28337, PMID: 36418241 PMC10099828

[B8] Huang Z. Hu B. Li J. Feng M. Wang Z. Huang F. . (2024). Metagenomic versus targeted next-generation sequencing for detection of microorganisms in bronchoalveolar lavage fluid among renal transplantation recipients. Front. Immunol. 15. doi: 10.3389/fimmu.2024.1443057, PMID: 39253087 PMC11381253

[B9] Jiang Y. Huang X. Zhou H. Wang M. Wang S. Ren X. . (2025a). Clinical characteristics and prognosis of patients with severe pneumonia with pneumocystis jirovecii colonization. CHEST 167, 54–66. doi: 10.1016/j.chest.2024.07.140, PMID: 39053646

[B10] Jiang Y. Huang X. Zhou H. Wang M. Wang S. Ren X. . (2025b). Clinical characteristics and prognosis of patients with severe pneumonia with pneumocystis jirovecii colonization: A multicenter, retrospective study. Chest 167, 54–66. doi: 10.1016/j.chest.2024.07.140, PMID: 39053646

[B11] Li D. Ding L. Luo J. Li Q.-G. (2023). Prediction of mortality in pneumonia patients with connective tissue disease treated with glucocorticoids or/and immunosuppressants by machine learning. Front. Immunol. 14. doi: 10.3389/fimmu.2023.1192369, PMID: 37304293 PMC10248221

[B12] Liu F. Zhuang Y. Huang X. Papazian L. Cai H. Shao H. . (2025). The Landscape of lower respiratory tract herpesviruses in severe pneumonia patients: a multicenter, retrospective study with prospective validation. Crit. Care 29, 254. doi: 10.1186/s13054-025-05496-3, PMID: 40542368 PMC12180245

[B13] Martin-Loeches I. Reyes L. F. Rodriguez A. (2025). Severe community-acquired pneumonia (sCAP): advances in management and future directions. Thorax, 80, 222296. doi: 10.1136/thorax-2024-222296, PMID: 40360263

[B14] Miao Q. Ma Y. Wang Q. Pan J. Zhang Y. Jin W. . (2018). Microbiological diagnostic performance of metagenomic next-generation sequencing when applied to clinical practice. Clin. Infect. Dis. 67, S231–S240. doi: 10.1093/cid/ciy693, PMID: 30423048

[B15] Nair G. B. Niederman M. S. (2021). Updates on community acquired pneumonia management in the ICU. Pharmacol. Ther. 217, 107663. doi: 10.1016/j.pharmthera.2020.107663, PMID: 32805298 PMC7428725

[B16] Niederman M. S. Torres A. (2022). Severe community-acquired pneumonia. Eur. Respir. Rev. 31, 220123. doi: 10.1183/16000617.0123-2022, PMID: 36517046 PMC9879347

[B17] Park J. H. Hong S.-B. Huh J. W. Jung J. Kim M. J. Chong Y. P. . (2024). Severe human parainfluenza virus community- and healthcare-acquired pneumonia in adults at tertiary hospital, Seoul, South Korea 2010–2019. Emerg. Infect. Dis. 30, 1088–1095. doi: 10.3201/eid3006.230670, PMID: 38781685 PMC11138994

[B18] Peetermans M. Matheeussen V. Moerman C. De Rydt F. Thieren S. Pollet E. . (2024). Clinical and molecular epidemiological features of critically ill patients with invasive group A Streptococcus infections: a Belgian multicenter case-series. Ann. Intensive Care 14, 19. doi: 10.1186/s13613-024-01249-7, PMID: 38286885 PMC10825083

[B19] Qin Q. Yu H. Zhao J. Xu X. Li Q. Gu W. . (2024). Machine learning-based derivation and validation of three immune phenotypes for risk stratification and prognosis in community-acquired pneumonia: a retrospective cohort study. Front. Immunol. 15. doi: 10.3389/fimmu.2024.1441838, PMID: 39114653 PMC11303239

[B20] Ramirez J. A. Musher D. M. Evans S. E. Dela Cruz C. Crothers K. A. Hage C. A. . (2020). Treatment of community-acquired pneumonia in immunocompromised adults. Chest 158, 1896–1911. doi: 10.1016/j.chest.2020.05.598, PMID: 32561442 PMC7297164

[B21] Reichel F. Tesch F. Berger S. Seifert M. Koschel D. Schmitt J. . (2024). Epidemiology and risk factors of community-acquired pneumonia in patients with different causes of immunosuppression. Infection 52, 2475–2486. doi: 10.1007/s15010-024-02314-w, PMID: 38935248 PMC11621203

[B22] Reyes L. F. Sanabria-Herrera N. Nseir S. Ranzani O. T. Povoa P. Diaz E. . (2025). Nosocomial lower respiratory tract infections in patients with immunosuppression: a cohort study. Ann. Intensive Care 15. doi: 10.1186/s13613-025-01462-y, PMID: 40328994 PMC12055687

[B23] Sajda P. (2006). Machine learning for detection and diagnosis of disease. Annu. Rev. Biomed. Eng 8, 537–565. doi: 10.1146/annurev.bioeng.8.061505.095802, PMID: 16834566

[B24] Sayson S. G. Ashbaugh A. Porollo A. Smulian G. Cushion M. T. (2024). *Pneumocystis murina* promotes inflammasome formation and NETosis during *Pneumocystis* pneumonia. mBio 15, e01409–e01424. doi: 10.1128/mbio.01409-24, PMID: 38953359 PMC11323544

[B25] Simner P. J. Miller S. Carroll K. C. (2018). Understanding the promises and hurdles of metagenomic next-generation sequencing as a diagnostic tool for infectious diseases. Clin. Infect. Dis. 66, 778–788. doi: 10.1093/cid/cix881, PMID: 29040428 PMC7108102

[B26] Song W. Yang Q. Lv H. Lv Y. Jiang Y. Qu J. . (2025). Prospective multicenter study identifying prognostic biomarkers and microbial profiles in severe CAP using BALF, blood mNGS, and PBMC transcriptomics. Sci. Rep. 15, 16252. doi: 10.1038/s41598-025-00812-x, PMID: 40346111 PMC12064704

[B27] Vidaur L. Guridi A. Leizaola O. Marin J. Rello J. Sarasqueta C. . (2025). Respiratory dysbiosis as prognostic biomarker of disease severity for adults with community-acquired pneumonia requiring mechanical ventilation. Pneumonia 17, 10. doi: 10.1186/s41479-025-00163-1, PMID: 40320531 PMC12051328

[B28] Wu X. Sun T. He H. Xing L. Cheng Z. Geng S. . (2025). Effect of metagenomic next-generation sequencing on clinical outcomes of patients with severe community-acquired pneumonia in the ICU. CHEST 167, 362–373. doi: 10.1016/j.chest.2024.07.144, PMID: 39067508

[B29] Xie K. Guan S. Kong X. Ji W. Du C. Jia M. . (2024). Predictors of mortality in severe pneumonia patients: a systematic review and meta-analysis. Syst. Rev. 13, 210. doi: 10.1186/s13643-024-02621-1, PMID: 39103964 PMC11302088

[B30] Xu Z. Guo K. Chu W. Lou J. Chen C. (2022). Performance of machine learning algorithms for predicting adverse outcomes in community-acquired pneumonia. Front. Bioeng Biotechnol. 10. doi: 10.3389/fbioe.2022.903426, PMID: 35845426 PMC9278327

[B31] Xu J. Zhong L. Shao H. Wang Q. Dai M. Shen P. . (2023a). Incidence and clinical features of HHV-7 detection in lower respiratory tract in patients with severe pneumonia: a multicenter, retrospective study. Crit. Care 27, 248. doi: 10.1186/s13054-023-04530-6, PMID: 37353839 PMC10290302

[B32] Xu J. Zhou P. Liu J. Zhao L. Fu H. Han Q. . (2023b). Utilizing metagenomic next-generation sequencing (mNGS) for rapid pathogen identification and to inform clinical decision-making: results from a large real-world cohort. Infect. Dis. Ther. 12, 1175–1187. doi: 10.1007/s40121-023-00790-5, PMID: 36988865 PMC10147866

