# Supplementary material for: Identification of subtypes and construction of a predictive model for novel subtypes in severe community-acquired pneumonia based on clinical metagenomics: a multicenter, retrospective cohort study
Source: Front Cell Infect Microbiol. 2025 Dec 9;15:1676502. doi: 10.3389/fcimb.2025.1676502 (PMC12722990; doi:10.3389/fcimb.2025.1676502)
Supplement: Supplementary file 1 [file Table1.doc]

**Table S1. Variance Inflation Factor of Independent Variables.**

|  | **VIF** | **tolerance** |
| --- | --- | --- |
| Intravenous Corticosteroids | 1.095 | 0.913 |
| Immunosuppression | 1.545 | 0.647 |
| CKD | 1.16 | 0.862 |
| HM | 1.113 | 0.898 |
| CTD | 1.131 | 0.884 |
| Transplantation | 1.324 | 0.755 |
| Klebsiella | 1.055 | 0.948 |
| Pneumocystis | 1.253 | 0.798 |
| EBV | 1.101 | 0.908 |
| CMV | 1.233 | 0.811 |

HM, Hematologic malignancy; CTD, Connective tissue disease; CKD, Chronic kidney disease; CMV, Cytomegalovirus; EBV, Epstein-Barr virus.
